# Sex-Related Differences in Show-Jumping Performance of Retired Thoroughbred Racehorses in Relation to the Interval Since Race Retirement

**DOI:** 10.3390/ani16040562

**Published:** 2026-02-11

**Authors:** M. Naito, S. Nishihata, T. Amano

**Affiliations:** 1 Laboratory of Animal Genetics, Graduate School of Dairy Science, Rakuno Gakuen University, 582 Midorimachi, Bunkyodai, Ebetsu 069-8501, Hokkaido, Japan; s22531018@stu.rakuno.ac.jp; 2 Laboratory of Animal Genetics, Department of Sustainable Agriculture, College of Agriculture, Food and Environment Sciences, Rakuno Gakuen University, 582 Midorimachi, Bunkyodai, Ebetsu 069-8501, Hokkaido, Japan; s22351233@stu.rakuno.ac.jp

**Keywords:** ex-racehorses, show jumping transition, effects of horse sex, Bayesian linear mixed models, performance determinants

## Abstract

To investigate the factors affecting the utilization of retired Thoroughbred racehorses in equestrian disciplines, Bayesian linear mixed models were separately fitted using rank, round time, and obstacle faults from show-jumping competitions restricted to retired Thoroughbred racehorses as dependent variables, with the interaction between horse sex and the interval from race retirement to competition (as a proxy for transition training to show-jumping) as a fixed effect. When the interval was short (≤1 year), the estimated marginal mean of rank was statistically significantly lower in stallions (0.26) than in mares (0.41) and geldings (0.39). However, ranking improved with longer intervals in all sexes, with the greatest improvement observed in stallions, and the significant sex-related differences disappeared at the 3-year interval, suggesting an effect of transition training on ranking. Round time improved significantly with longer intervals in all sexes, consistent with the ranking pattern; significant improvement in obstacle faults was observed only in stallions and geldings. The explanatory power of the models, including major random effects, rider, horse ability, sire and affiliation after retirement, was moderate (conditional R^2^: 0.40–0.65), whereas that of the fixed effects was small (marginal R^2^: 0.02–0.07), indicating the multifactorial nature of success in competition.

## Simple Summary

Because racehorses retire from racing at a young age, their subsequent utilization often requires a transition to equestrian disciplines. To clarify the factors affecting this transition process, we analyzed performance in show-jumping competitions restricted to retired Thoroughbred racehorses using a Bayesian linear mixed model, focusing on the effects of horse sex and the interval between race retirement and participation in competitions (used as a proxy for the period of transition training). Stallions performed worse than mares and geldings when the interval was short. However, performance improved with longer intervals across all sexes, particularly in stallions, and clear sex-related differences diminished at longer intervals, suggesting an effect of prolonged transition training. Although the fixed effects, with the sex and interval as the primary factors, accounted for only 2–7% of the performance variance, random effects, including horse-specific ability, rider, sire, and affiliation, accounted for 40–65% of the performance variance, highlighting the multifactorial nature of success in competition. In summary, our findings suggest that a sufficiently long interval, which may correspond to the period of transition training, is important for improving jumping competition performance regardless of sex. Moreover, factors other than the interval also contributed to performance.

## 1. Introduction

Most Thoroughbred racehorses complete their racing careers and retire around the age of 5 years. However, given the long lifespan of horses—approximately 25 years—new uses must be found for many of them [1,2]. A common option is to employ them in equestrian competitions such as dressage and show jumping after transition training (retraining) [2,3]. To investigate the factors affecting the success of this transition, previous studies have examined how racing performance and behavioral characteristics immediately after retirement are associated with subsequent suitability for equestrian use [3,4]. However, the ability of retired racehorses to perform successfully in these equestrian competitions is also likely affected by a variety of factors, including age, sex, and the duration of retraining after retirement. Clarifying the extent to which each factor affects competitive performance could provide valuable insights that would help promote the effective use of retired racehorses through the development of more refined training policies and rider–horse pairing strategies.

Previous analyses of equestrian competition performance, including show jumping, have reported that stallions and geldings outperform mares or, alternatively, that stallions perform better than both mares and geldings [5,6,7], with this tendency being stronger in more difficult competitions [5,6]. Although these findings are useful in the development of training and competition strategies, most of the studied populations consisted of breeds produced for equestrian competitions rather than Thoroughbreds used for racing, and several previous reports have noted performance differences among breeds [7,8,9]. In addition, racehorses were originally bred and trained exclusively for racing; thus, such findings may not necessarily apply when retired racehorses are retrained for equestrian competition.

To clarify the factors affecting the successful transition of retired Thoroughbred racehorses to equestrian disciplines, performance data from competitions restricted to retired Thoroughbred racehorses need to be examined. In Japan, such data are available through the Retired Racehorse Cup (RRC), a nationwide equestrian competition established in 2018 to promote the use of retired Thoroughbred racehorses for equestrian purposes. In this study, we fitted separate multivariable Bayesian linear mixed models for rank, round time, and obstacle faults in RRC show-jumping competitions. Each model included horse sex, age, and the time between retirement from racing and competition entry (defined as the interval, assumed to represent the retraining period) as covariates, with rider, rider–horse pair, individual horse ability (horse), sire, affiliation after retirement, competition year, and venue as random effects.

## 2. Materials and Methods

### 2.1. Overview of the Competition and Dependent Variables

The RRC qualifying competitions followed the Japan Equestrian Federation “Small Obstacle B” (90 cm class) rules and consisted of a course with 11 obstacles, divided into six in round 1 and five in round 2. Rankings were primarily determined by the total number of obstacle faults accumulated across both rounds, and in the case of ties, the round time in round 2 was used to determine placement. Further details on the RRC competition format, rules, and eligibility criteria are available from the official RRC website (in Japanese): https://jouba.nrca.or.jp/rrc/ (accessed on 6 January 2026).

In this study, the dependent variables included ranking, round time in the first and second rounds (round time 1 and round time 2, respectively), and net obstacle faults in each round, excluding time penalties (obstacle faults 1 and obstacle faults 2, respectively). Rankings at each venue within each competition were transformed using the following formula: 1 − (rank − 1)/(max (rank) − 1). Each qualifying competition had at least two records.

Round time values were log-transformed, and the resulting estimated marginal means (EMMs) were back-transformed using the exponential function.

Early RRC competitions had no age restriction, and a two-year-old horse competed once; however, for animal welfare reasons, participation was later limited to horses aged three years or older.

### 2.2. Data Overview and Descriptive Statistics

Between 2018 and 2024, data were collected from a total of 76 qualifying RRC competitions held annually at a cumulative total of 20 venues across Japan. Records for rider–horse pairs that were withdrawn or eliminated due to falls or refusals, as well as 36 records from 15 geldings for which sex changed from stallion to gelding during the study period, were excluded, resulting in 1951 records for analysis (Table S1).

The 1951 records analyzed in this study included 860 horses (38 stallions, 228 mares, and 594 geldings), 534 riders, and 1190 rider–horse pairs, of which 369 horses, 319 rider–horse pairs, and 376 riders participated more than once (Figure S1A,B). Figure S1C–F present the number of rider–horse pairs per competition year, the mean age of horses in each competition year, the mean transformed rank in each competition year, and the number of rider–horse pairs in each interval category, respectively.

In the first year of the competition (2018), only one round was held under conditions comparable to those of round 2, which was therefore treated as the second round in this study. Accordingly, analyses of the first round were conducted using 1831 records, excluding those from the year 2018. For the obstacle-fault analyses, net obstacle faults were calculable only when time-penalty values were available (see Materials and Methods, Section 2.1); therefore, records lacking time-penalty information were excluded. As a result, 893 first-round records (2019, 2021, 2023, and 2024) and 1017 second-round records (2018, 2019, 2021, 2023, and 2024) were available for the obstacle-fault analyses (four and five competition years, respectively).

In the analyses of the sex × interval and sex × age interactions for transformed rank, the mean transformed rank ± SD was calculated for each sex, for each interval category and age group, and for each sex within each interval category and age group. Differences among groups were tested using Kruskal–Wallis tests, followed by pairwise Wilcoxon tests with Holm correction.

### 2.3. Bayesian Linear Mixed Model Analysis

Because the competition dataset includes many grouping levels (e.g., horse, rider, and rider–horse pair), with sparse observations per level, we used Bayesian linear mixed models. Bayesian estimation with appropriate priors provides stable variance estimates in such sparse hierarchical structures [10].

In the Bayesian linear mixed model analysis, horse sex was included as the primary effect of interest, with interval and age included as covariates. Sex was categorized as stallion, mare, or gelding. Interval was defined as the time between retirement from racing (i.e., the date of the final race) and competition entry. Because the RRC targeted only horses within 3 years of retirement (deregistration from the racing registry), interval was classified as ≤1 year, >1 and ≤2 years (2 years), and >2 and ≤3 years (3 years). Age was categorized as 2–5 years, 6–7 years, 8–9 years, and 10–15 years. The RRC also accepted entries from horses that had been registered as racehorses but retired without participating in any races; therefore, the interval for these horses was undefined due to the lack of data regarding the final race date, so these horses were classified as the NI (non-interval) group. The ≤1 year, 2 years, 3 years, and NI groups included 754, 615, 488, and 94 records, respectively. The 2–5 years, 6–7 years, 8–9 years, and 10–15 years age groups included 705, 648, 427, and 171 records, respectively.

**Figure 1:**
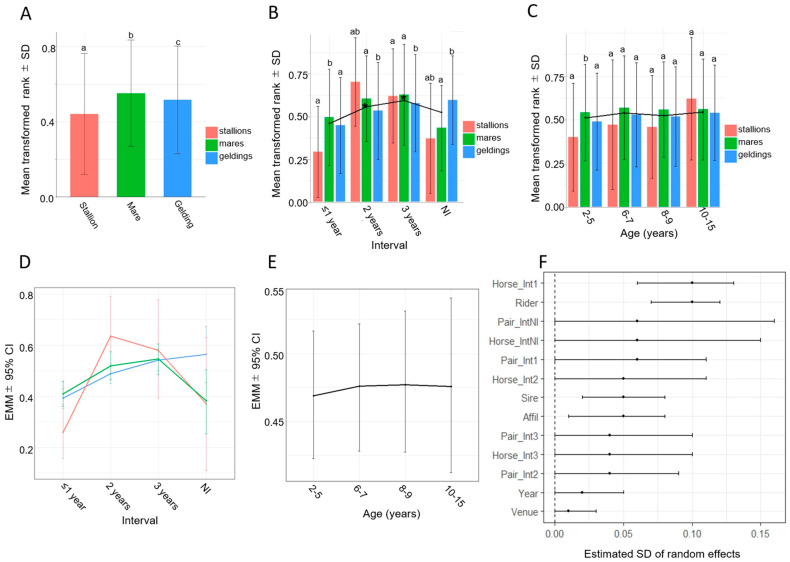
Rank-related results of descriptive analyses and Bayesian linear mixed-model analyses. (**A**) Mean transformed rank ± SD of stallions, mares, and geldings. (**B**) Mean transformed rank ± SD of stallions, mares, and geldings by interval between retirement from racing and participation in jumping competition (≤1 year, 2 years, 3 years, and the NI group, representing horses with no information regarding the interval). (**C**) Mean transformed rank ± SD of stallions, mares, and geldings by age group (2–5 years, 6–7 years, 8–9 years, and 10–15 years). (**D**) Estimated marginal means (EMMs) ± 95% credible intervals (CIs) for the interaction between sex and interval for transformed rank. (**E**) EMMs ± 95% CIs for transformed rank by age group. (**F**) Estimated SDs of random effects in the rank model. Each point represents the posterior mean, and horizontal lines indicate the 95% CIs. Abbreviations: Rider, rider; Pair_Int1y, random slope for rider–horse pair at the ≤1-year interval; Pair_Int2y, random slope for rider–horse pair at the 2-year interval; Pair_Int3y, random slope for rider–horse pair at the 3-year interval; Pair_IntNI, random slope for rider–horse pair in the NI group; Horse_Int1y, random slope for horse at the ≤1-year interval; Horse_Int2y, random slope for horse at the 2-year interval; Horse_Int3y, random slope for horse at the 3-year interval; Horse_IntNI, random slope for horse in the NI group; Sire, sire; Affil, affiliation after retirement; Year, competition year; Venue, competition venue. In panels (**A**–**D**), red, green, and blue bars or lines represent stallions, mares, and geldings, respectively. In panels (**A**–**C**), bars with different letters (a–c) indicate significant differences (*p* < 0.05). In panels (**B**,**C**), asterisks on the solid line indicate significant differences between specific interval or age groups (*p* < 0.05).

In the analysis of fixed effects, it was not feasible to simultaneously include interactions between sex and both covariates (age and interval) because of computational resource limitations. However, descriptive statistics suggested that sex-specific trends across interval categories diverged more than sex-specific trends across age categories, indicating that the sex × interval interaction was more salient than the sex × age interaction (Figure 1B). Furthermore, when rank was set as the dependent variable and models were preliminarily fitted with either a sex × age or a sex × interval interaction, statistically meaningful effects were observed only in the model including the sex × interval interaction. Therefore, the interaction between sex and interval was incorporated in the present analysis.

Random effects were specified for rider (534 levels), horse (860 levels), sire (210 levels), affiliation after retirement (defined as the facility responsible for housing and equestrian training the horses after retirement from racing; 259 levels), competition year (2018–2024, 7 levels), and venue (20 venues across Japan; 20 levels). To account for the combined effect of rider and horse, rider–horse pair (1190 levels) was additionally included (Table S2). Because interval was assumed to correspond to the retraining period and could substantially influence changes in performance, random slopes for interval were specified for the horse and for the rider–horse pair. For the analyses of round time and obstacle faults, random slopes for interval were excluded for the horse and rider–horse pair to reduce computational burden. The full model formula for the transformed-rank analysis is provided below (where “Sex * Interval” denotes the main effects of sex and interval and their interaction). For round time and obstacle faults, the same fixed-effects structure was used, but without the interval random slopes for horse and rider–horse pair:

Transformed rank ~ Sex * Interval + Age + (1 | Rider) + (1 + Interval || Rider–horse pair) + (1 + Interval || Horse) + (1 | Sire) + (1 | Affiliation after retirement) + (1 | Competition year) + (1 | Venue)

In this analysis, we constructed a model assuming a Student-*t* error distribution within a Bayesian framework. However, a Gaussian distribution was applied to the data for obstacle faults 1 and 2 because obstacle-fault scores reflect an underlying continuous process, and because the negative binomial and zero-inflated models failed to converge due to the large number of zeros [11].

The model was built using the brms package [12,13], and sampling was performed using the probabilistic programming language Stan [14]. Default priors provided by brms were used: a flat prior was set for the fixed-effects coefficients, a Student-*t* distribution (df = 3, mean = 0, scale = 10) for the intercept, a half–Student-*t* distribution (df = 3, scale = 10) for the residual scale and the SD of random effects, and a Gamma (2, 0.1) distribution for the degrees of freedom parameter of the Student-*t* distribution. These priors were automatically adjusted according to the distribution of the dependent variable.

Estimation was performed using four chains, each running for 5000 iterations (2500 for warm-up). Convergence was confirmed using the R-hat statistic [15] and effective sample size (Bulk ESS and Tail ESS) [16]. All parameters showed R-hat values < 1.01 and sufficiently large ESS values (typically >1000), with no divergent transitions or other sampler warnings observed. Model fit was assessed using posterior predictive interval coverage for all dependent variables [17,18]. The coverage values were close to, or slightly higher than, the nominal levels, suggesting mildly conservative predictive intervals and indicating no major lack of fit (Table S3). The model’s explanatory power was assessed using Bayesian R^2^ (conditional and marginal) [19]. In addition, to compare the relative importance of random effects, approximate R^2^ values were derived by distributing the difference between the conditional and marginal Bayesian R^2^ in proportion to the posterior variance components of random effects that showed statistically meaningful contributions.

The Bayesian linear mixed model used in this study can be expressed as follows:

Y = Xβ+ Zb + ε

where *Y* represents the dependent variable, *X* and *Z* represent the design matrices for the fixed and random effects, respectively, *β* represents the vector of fixed-effect coefficients, *b* represents the vector of random effects assumed to follow *b* ~ *N* (0, Σb), and *ε* represents the residual error assumed to follow *ε* ~ Student-*t*(0, σ, ν).

For fixed effects, the effect of each factor was interpreted using the posterior mean of the EMMs with 95% credible intervals (CIs). EMM values were calculated using the R4.2.2 package “emmeans” according to the following equation:

$$\mathrm{EMM}=E\left(Y|X_{ref}\right)=X_{ref}\beta .$$

Here, *Y* denotes the dependent variable (e.g., rank), and the expression *E*(*Y* | *X_ref_*) represents the expected value of *Y* under a specific combination of factor levels, which corresponds to the EMM. The vector *β* consists of the regression coefficients of the fixed effects estimated by the model, and the linear predictor for the reference condition is obtained by multiplying *X_ref_* (which defines the specified levels of the fixed factors) by *β*. EMMs were estimated for sex, age, and the interaction between sex and interval. Differences between EMMs were evaluated using the posterior distribution of their contrasts and were considered statistically meaningful when the 95% CI of the contrast did not include zero. For random effects, the estimated SDs were reported together with their 95% CIs. A level was considered to show a meaningful effect if the lower bound of the 95% CI was >0.

### 2.4. Analysis of Transformed-Rank Trends in Horses with Records at Multiple Interval Categories

In this study, an improvement in transformed rank was observed with increasing interval, possibly reflecting the effect of a longer retraining period. However, this improvement may only reflect selective withdrawal of poorly performing horses from subsequent competitions, with no effect of retraining. If this were the case, performance would not be expected to improve within the same horses over time. To address this possibility, changes in transformed rank across interval categories were examined in horses with records at both the ≤1-year and 2-year intervals (stallions: 1 horse, 8 records; mares: 47 horses, 253 records; geldings: 106 horses, 373 records), as well as in horses with records at all interval categories of ≤1 year, 2 years, and 3 years (mares: 28 horses, 255 records; geldings: 49 horses, 266 records).

The Bayesian linear mixed model used for this analysis included interval, sex, and age as fixed effects, with horse included as a random effect to account for repeated measurements within individuals. The model formula was as follows:

Transformed rank ~ Interval + Sex + Age + (1 | Horse)

EMMs were obtained from the model, and differences between EMMs across intervals were evaluated using the posterior distribution of their contrasts. Statistical significance was inferred when the 95% CI of the contrast did not include zero.

No generative artificial intelligence (GenAI) tools were used in this study.

## 3. Results

### 3.1. Descriptive Statistics of Transformed Rank by Sex, Interval, and Age, and Interaction Patterns Across Interval and Age

In the comparison of mean transformed rank among sexes, performance was highest in mares, followed by geldings and stallions, with statistically significant differences between each pair (*p* < 0.05; Figure 1A). Across interval categories, the mean transformed rank tended to increase with longer intervals in all sexes, and pairwise comparisons revealed significant differences among all interval groups (*p* < 0.05; Figure 1B). Within interval categories, mares generally performed best, followed by geldings and stallions; however, the sex effect differed across interval categories. Specifically, the mean transformed rank of mares was significantly better than that of the other sexes at the ≤1-year interval and better than that of geldings thereafter (*p* < 0.05; Figure 1B). In the NI group, geldings achieved significantly better mean transformed ranks than the other sexes (*p* < 0.05; Figure 1B). Across age groups, no significant differences were detected in the overall mean transformed rank (*p* > 0.05; Figure 1C). However, within each age group, the mean transformed rank generally tended to follow the order mares > geldings > stallions; however, a statistically significant difference was observed only in the 2–5-year group, where mares performed significantly better than the other sexes (*p* < 0.05; Figure 1C). The medians and ranges of transformed rank, round time (rounds 1 and 2), and obstacle faults (rounds 1 and 2), stratified by sex, interval, age, and number of competitions, are provided in Table S4.

### 3.2. Analysis Using Bayesian Linear Mixed Models with Transformed Rank as the Dependent Variable

Stallions showed worse rankings than the other sexes at the ≤1-year interval, with statistically significant differences observed between stallions and both mares and geldings. However, no significant sex-related differences in rank were observed at either the 2-year or the 3-year interval (Figure 1D, Table 1). Across intervals, rank improved with longer intervals in all sexes, with statistically significant differences observed between the ≤1-year interval and both the 2-year and 3-year intervals in stallions and mares, and between all interval categories in geldings (Figure 1D, Table 2). In the NI group, geldings tended to perform better than the other sexes, but a statistically significant difference was observed only between geldings and mares (Figure 1D, Table 1).

No statistically significant effect of age was observed on transformed rank (Figure 1E). Random effects that were judged significant were rider, horse at the ≤1-year interval, sire, and affiliation after retirement (Figure 1F), with approximate R^2^ values of 0.15, 0.15, 0.05, and 0.05, respectively.

### 3.3. Transformed-Rank Trends in Horses with Records at Multiple Interval Categories

**Figure 2:**
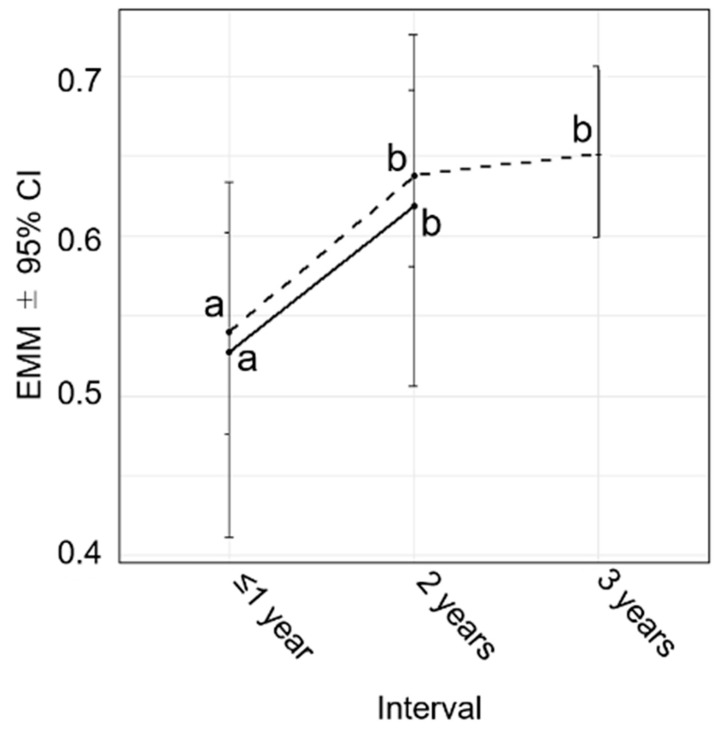
Transformed rank trends in horses with records at multiple interval categories. Estimated marginal means (EMMs) ± 95% credible intervals (CIs) of transformed rank are shown for horses with records at both the ≤1-year and 2-year intervals (solid line) and for horses with records at all three interval categories (≤1 year, 2 years, and 3 years; dashed line). Points represent posterior means of the EMMs, and vertical bars indicate 95% CIs. Different letters (a and b) indicate significant differences between interval categories within each line.

In the Bayesian linear mixed-model analysis restricted to horses with records at both the ≤1-year and 2-year intervals, transformed rank was significantly higher at the 2-year interval than at the ≤1-year interval (Figure 2). Similarly, in the analysis restricted to horses with records across all three interval categories (≤1 year, 2 years, and 3 years), transformed rank was significantly higher at both the 2-year and 3-year intervals than at the ≤1-year interval (Figure 2).

### 3.4. Analysis Using Bayesian Linear Mixed Models with Round Time 1 as the Dependent Variable

**Figure 3:**
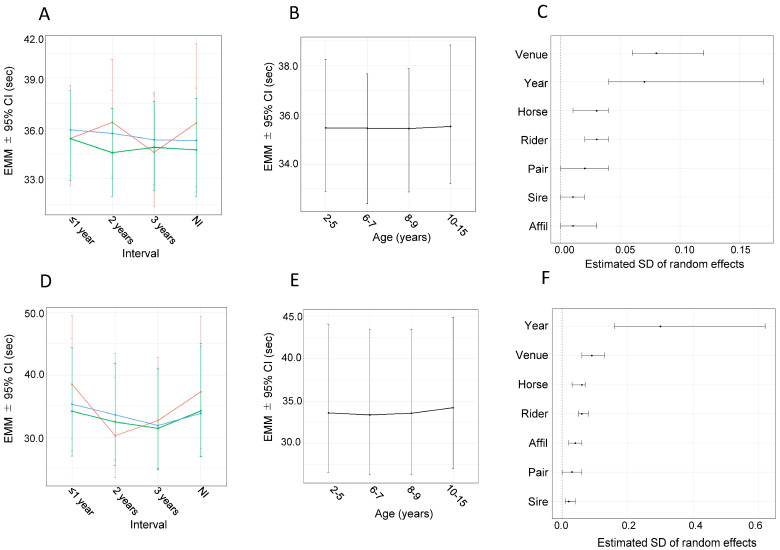
Round time–related results of Bayesian linear mixed-model analyses. (**A**) Estimated marginal means (EMMs) ± 95% credible intervals (CIs) for the interaction between sex and interval for round time 1. (**B**) EMMs ± 95% CIs for round time 1 by age group. (**C**) Estimated SDs of random effects in the round time 1 model. Each point represents the posterior mean, and horizontal lines indicate the 95% CIs. (**D**) EMMs ± 95% CIs for the interaction between sex and interval for round time 2. (**E**) EMMs ± 95% CIs for round time 2 by age group. (**F**) Estimated SDs of random effects in the round time 2 model. Each point represents the posterior mean, and horizontal lines indicate the 95% CIs. In panels (**A**,**D**), red, green, and blue lines represent stallions, mares, and geldings, respectively. In panels (**C**,**F**), abbreviations are as defined in the legend for Figure 1F.

For round time 1, sex-related differences within interval categories were generally not evident; however, mares showed significantly shorter round times than geldings at the 2-year interval (Figure 3A, Table 1). In addition, round time 1 decreased only slightly with increasing intervals, and statistically significant reductions were observed in mares between the ≤1-year and 2-year intervals and in geldings between the ≤1-year and 3-year intervals, whereas no significant interval-related change was detected in stallions (Figure 3A, Table 2). In the NI group, round time 1 did not differ among sexes (Figure 3A, Table 1).

No statistically significant effect of age was observed on the round time 1 (Figure 3B). Random effects that were judged significant were venue, year, horse, and rider (Figure 3C), with approximate R^2^ values of 0.21, 0.16, 0.03, and 0.03, respectively.

### 3.5. Analysis Using Bayesian Linear Mixed Models with Round Time 2 as the Dependent Variable

Round time 2 improved with longer intervals in all sexes, with statistically significant differences observed between most interval categories, except for the comparison between the 2- and 3-year intervals in stallions and mares (Figure 3D, Table 2). Within interval categories, significant sex-related differences were observed at the ≤1-year interval, with mares showing the shortest round times, followed by geldings and stallions. At the 2-year interval, round time was shorter in stallions and mares than in geldings, whereas no significant sex-related differences were observed at the 3-year interval (Figure 3D, Table 1). Round time 2 did not differ between sexes in the NI group (Figure 3D, Table 1).

No statistically significant effect of age on round time 2 was observed (Figure 3E). Random effects that were judged significant were year, venue, rider, horse, affiliation, and sire (Figure 3F), with approximate R^2^ values of 0.51, 0.05, 0.02, 0.02, 0.01, and 0.00, respectively.

### 3.6. Analysis Using Bayesian Linear Mixed Models with Obstacle Faults 1 as the Dependent Variable

**Figure 4:**
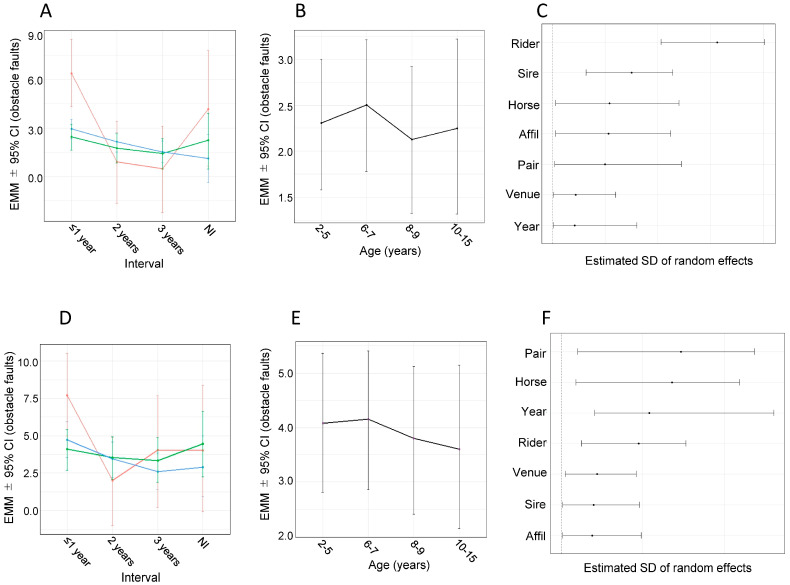
Obstacle faults–related results of Bayesian linear mixed-model analyses. (**A**) Estimated marginal means (EMMs) ± 95% credible intervals (CIs) for the interaction between sex and interval for obstacle faults 1. (**B**) EMMs ± 95% CIs for obstacle faults 1 by age group. (**C**) Estimated SDs of random effects in the obstacle faults 1 model. Each point represents the posterior mean, and horizontal lines indicate the 95% CIs. (**D**) EMMs ± 95% CIs for the interaction between sex and interval for obstacle faults 2. (**E**) EMMs ± 95% CIs for obstacle faults 2 by age group. (**F**) Estimated SDs of random effects in the obstacle faults 2 model. Each point represents the posterior mean, and horizontal lines indicate the 95% CIs. In panels (**A**,**D**), red, green, and blue lines represent stallions, mares, and geldings, respectively. In panels (**C**,**F**), abbreviations are as defined in the legend for Figure 1F.

For obstacle faults 1, stallions performed worse than the other sexes at the early stage of the interval; statistically significant differences were observed between stallions and both mares and geldings at the ≤1-year interval (Figure 4A, Table 1). With longer intervals, obstacle faults decreased in stallions and geldings, leading to the disappearance of sex-related differences at the 2- and 3-year intervals, whereas no significant interval-related change was observed in mares (Figure 4A, Table 2). Obstacle faults 1 did not differ among sexes in the NI group (Figure 4A, Table 1).

No statistically significant effect of age was observed on the obstacle faults 1 (Figure 4B). Random effects that were judged significant were rider and sire (Figure 4C), with approximate R^2^ values of 0.27 and 0.06, respectively.

### 3.7. Analysis Using Bayesian Linear Mixed Models with Obstacle Faults 2 as the Dependent Variable

For obstacle faults 2, stallions performed worse than the other sexes at the ≤1-year interval, with statistically significant differences observed only between stallions and both mares and geldings (Figure 4D, Table 1). Across intervals, obstacle faults statistically decreased in stallions and geldings, and sex-related differences were not significant at the later interval, 3 years, whereas no significant interval-related change was observed in mares (Figure 4D, Table 2). Obstacle faults 2 did not differ among sexes in the NI group (Figure 4D, Table 1).

No statistically significant effect of age was observed on obstacle faults 2 (Figure 4E). All random effects were judged significant: rider–horse pair, horse, competition year, rider, venue, sire, and affiliation after retirement (Figure 4F), with approximate R^2^ values of 0.11, 0.10, 0.06, 0.05, 0.01, 0.01, and 0.01, respectively.

### 3.8. Explanatory Power of the Model

For all dependent variables, the conditional R^2^ values, which incorporate both fixed and random effects, ranged from 0.40 to 0.65, indicating a moderate level of explanatory power. By contrast, the marginal R^2^ values, reflecting fixed effects alone, ranged from 0.02 to 0.07, suggesting that most of the explanatory power of the models was attributable to random effects (Table 3).

## 4. Discussion

In this study, we used a Bayesian linear mixed model to analyze factors influencing show-jumping performance in retired Thoroughbred racehorses, with a focus on horse sex and the interval between race retirement and competition entry, which may correspond to the retraining period for show jumping. Mares and geldings outperformed stallions at short intervals after race retirement, whereas performance improved across all sexes as the interval increased, resulting in no clear sex-related differences at later intervals, consistent with an effect of the prolonged retraining period. On the other hand, fixed effects, with sex and interval as the primary factors, accounted for only a small portion (7%) of the variance in ranking, whereas random effects, including horse-specific ability, rider, sire, and affiliation after retirement as major contributors, accounted for 44% of the variation in ranking, highlighting the multifactorial nature of success in show jumping in retrained retired racehorses.

In the descriptive analysis of transformed rank, the overall mean transformed rank was highest in mares, followed by geldings, and lowest in stallions (Figure 1A), and the mean transformed rank increased with longer intervals in all sexes (Figure 1B). Mares performed better than geldings across all interval years, whereas stallions showed poorer performance at the ≤1-year interval but improved thereafter, with a significant increase (Figure 1B). These descriptive trends were largely retained in the Bayesian model-based analysis, with stallions exhibiting markedly worse transformed ranks than the other sexes at the ≤1-year interval (Figure 1D). While temperament and/or behavioral difficulties of stallions have been considered a cause of poorer performance at the early stage of training (corresponding to the ≤1-year interval in our study) [5], several studies have reported results different from ours, showing that stallions and geldings perform better than mares or that stallions outperform the other sexes [5,6,7]. These findings have been explained in those previous studies by the superior speed and jumping ability of stallions, which have been suggested to be associated with higher aerobic capacity [20,21] and greater explosive power [22,23]. One possible explanation for this discrepancy is differences in horse usage between our study and previous studies. Specifically, those studies focused exclusively on horses originally bred for equestrian purposes. In such populations, stallions may receive more intensive training than other sexes for evaluation as potential sires [24], which could enhance their performance—a situation that does not apply to retired Thoroughbred racehorses. On the other hand, fewer stallions were analyzed in our study compared with mares and geldings (Figure S1A), and this sample imbalance may have influenced the results. Furthermore, as stallions with performance problems are often gelded, the observed trend in stallions in our study may also reflect the characteristics of a subset of stallions that did not require gelding. However, with longer intervals, rankings improved in all sexes, with the most pronounced improvement observed in stallions, and the sex-related difference observed at the ≤1-year interval disappeared, highlighting the importance of transition training from racing to show jumping across sexes, particularly in stallions (Figure 1D).

Within the interval factor, the NI group comprised horses with no history of racing and therefore had no definable interval length. These horses were included in the model as a separate interval factor level for convenience. In the NI group, mares had a statistically higher rank than geldings (Figure 1D, Table 1), and this may reflect factors specific to horses with no racing experience, such as the absence of racing-related injuries. However, although horses in the NI group shared the common feature of having no racing experience, their training history, management conditions, and ability levels were likely more diverse than those of horses with racing experience. Such heterogeneity in backgrounds could obscure the effects of sex; as a result, it is difficult to draw clear conclusions about the characteristics of the NI group from the present results.

For the analysis of ranking, the conditional R^2^ was 0.44, whereas the marginal R^2^ was 0.07 (Table 3), indicating a substantially greater contribution of random effects than fixed effects, including sex and interval. To facilitate comparison of the relative importance of fixed effects and individual random factors, approximate R^2^ values were calculated for random effects with statistically significant contributions (Figure 1F) by distributing the difference between the conditional and marginal R^2^ (0.37) according to the proportion of variance explained by each factor: 0.15 for horse at the ≤1-year interval, 0.15 for rider, 0.05 for sire, and 0.05 for affiliation after retirement. Each of these values was roughly comparable to the contribution of the fixed effects primarily attributable to the interval (R^2^ = 0.07), potentially reflecting the retraining period. These results suggest that ranking was not determined by a single dominant factor but rather by multiple contributors, including the retraining period, individual horse ability, rider skill, genetic background, and post-retirement management environment.

The random-effects analysis of transformed rank showed that the horse-specific effect at the ≤1-year interval was the largest source of variation, suggesting that individual differences among horses strongly influenced ranking at the early stage of retraining (Figure 1F). However, beyond the first year after retirement, horse-specific effects were no longer prominent, indicating that with longer intervals, the relative contribution of individual horse effects decreased and that sufficient retraining time may attenuate the impact of initial individual differences. Moreover, rider effects were also substantial, indicating that rider skill contributed independently to variation in ranking. Sire and affiliation after retirement showed moderate but consistent effects, indicating that both genetic background and the post-retirement management environment contributed to ranking (Figure 1F).

In general, horses kept at equestrian facilities are routinely ridden and exercised on a daily basis as part of their regular management for competition. Therefore, the improvement in ranking observed with longer intervals in our study is primarily thought to reflect the effect of a longer retraining period. However, this improvement may only reflect selective withdrawal of poorly performing horses from subsequent competitions, with no effect of retraining. If this were the case, performance would not be expected to improve within the same horses over time. To evaluate this possibility, changes in transformed rank were examined using Bayesian linear mixed model analyses under two restricted conditions: (i) horses with records at both the ≤1-year and 2-year interval categories, and (ii) horses with records across all interval categories (≤1 year, 2 years, and 3 years). In both analyses, transformed rank increased significantly with increasing interval, suggesting that the observed improvement cannot be explained solely by selective withdrawal and is consistent with a substantial contribution of retraining (Figure 2).

For round time 1, interval-related trends within each sex were generally flat, and sex-related differences within interval categories were largely absent (Figure 3A; Table 1 and Table 2). This trend is consistent with the nature of round 1, in which only obstacle faults are considered for ranking, with no contribution from round time. The conditional R^2^ was 0.45, whereas the marginal R^2^ was 0.02, indicating that most of the model’s explanatory power could be attributed to the random effects rather than fixed effects, including sex and interval (Table 3). Among the random effects, significant contributions were observed for venue, competition year, horse and rider; however, the approximate R^2^ values were higher for venue and competition year (0.21 and 0.16, respectively), whereas those for horse and rider were only 0.03 (Figure 3C). These results suggest that round time 1 is more strongly influenced by competition-related environmental factors, such as weather conditions, than by rider skill and intrinsic horse ability, again consistent with the nature of round 1.

For round time 2, stallions performed significantly worse than mares and geldings at the ≤1-year interval. Performance then improved with longer intervals in all sexes, particularly in stallions, and the sex-related differences observed at the early stage were no longer evident at the 2- and 3-year intervals, suggesting an effect of a longer retraining period for all sexes (Figure 3D). However, despite these interval- and sex-related trends, the overall variation in round time 2 was driven predominantly by random effects rather than by fixed effects, including sex and interval: the conditional R^2^ was large (0.65), whereas the marginal R^2^ was only 0.04 (Table 3). The approximate R^2^ values showed a dominant contribution of competition year (R^2^ = 0.51), with only minor contributions from the other random effects, venue, horse and rider (R^2^ = 0.00–0.02). Unlike round time 1, round time 2 contributes directly to ranking; therefore, riders tend to attempt shortcuts to reduce time. In early RRC competitions, the course design allowed only highly skilled rider–horse pairs to use such shortcuts, whereas this option was later removed. This change likely resulted in a polarization of performance between pairs that could and could not exploit the shortcut, which may explain the strong effect of competition year.

For obstacle faults 1, stallions performed significantly worse than mares and geldings at the ≤1-year interval, but this disadvantage disappeared at longer intervals, suggesting a strong effect of retraining on stallions. Geldings also improved significantly with longer intervals, whereas mares showed only weak and non-significant improvement (Figure 4A; Table 1 and Table 2). This pattern could indicate that additional retraining has a limited effect on jumping performance in mares; however, it may also suggest that mares complete the necessary retraining at a very early stage within the ≤1-year interval category. Consistently, mares have been reported to perform better than stallions at the beginner level of show-jumping competitions [5,6]. On the other hand, despite these sex- and interval-related patterns, the overall variation in obstacle faults 1 was explained more by random effects than by fixed effects, including sex and interval: the conditional R^2^ was 0.40, whereas the marginal R^2^ was only 0.07 (Table 3). Among the random effects examined in this study, rider and sire showed clear contributions in the analysis of obstacle faults 1, with approximate R^2^ values of 0.27 and 0.06, respectively (Figure 4C; Table 3). In the first round, ranking is determined solely by obstacle faults, and course time is not considered. Therefore, the random effects identified in the analysis of obstacle faults 1, rider and sire, are expected to be more directly related to jumping performance than those found in obstacle faults 2.

For obstacle faults 2, stallions again performed significantly worse than mares and geldings at the ≤1-year interval, but this difference diminished with longer intervals and was no longer evident at the 2- and 3-year intervals, suggesting a clear effect of a prolonged retraining period on stallions. As observed for obstacle faults 1, geldings also improved significantly with longer intervals, whereas mares showed only weak and non-significant improvement (Figure 4D; Table 1 and Table 2), which may be attributable to the earlier completion of retraining in mares, as discussed above. Despite the clear sex- and interval-related patterns observed in the fixed-effect analysis, most of the variance in obstacle faults 2 was accounted for by random effects: the conditional R^2^ was 0.41, indicating moderate explanatory power, whereas the marginal R^2^ for the fixed effects of sex and interval was only 0.06 (Table 3). All random effects showed clear contributions; however, each of the corresponding approximate R^2^ values was relatively low (0.01–0.11) compared with those observed for obstacle faults 1 (rider = 0.27 and sire = 0.06), suggesting that a small but diverse array of factors contributes to obstacle faults 2. This pattern may be related to the fact that, unlike the first round, both obstacle faults and round time directly determine the ranking in the second round, resulting in a greater diversity of factors that can influence obstacle faults (Figure 4F).

In summary, to explore the factors affecting the transition of retired racehorses to equestrian disciplines, we analyzed show-jumping performance in retired Thoroughbred racehorses using a Bayesian linear mixed model, focusing on horse sex, age and the interval between retirement and competition entry. Our findings suggest that a sufficiently long interval, which may reflect the transition training period, is important for improving jumping performance regardless of sex, while factors other than the interval also contributed to performance.

## 5. Conclusions

This study examined performance in jumping competitions exclusively for retired Thoroughbred racehorses, focusing on the effects of sex, age, and the interval from retirement to competition participation while accounting for multiple random effects. Performance improved markedly with longer intervals, which may correspond to the transition training period for show jumping, across all sexes after retirement, with a particularly strong effect observed in stallions. However, fixed effects (sex and interval) explained only a small proportion of variance (marginal R^2^ = 0.02–0.07), whereas the overall explanatory power was moderate when random effects were included (conditional R^2^ = 0.40–0.65), reflecting contributions from rider, horse (particularly at the early stage of the interval), sire, and affiliation after retirement. Our findings suggest that not only prolonged retraining but also other factors, including rider skill, horse ability at the early stage of transition training, horse pedigree, and the management environment after retirement, contribute to the use of retired Thoroughbred racehorses in show-jumping competitions to a similar extent.

## Figures and Tables

**Table 1:** Pairwise comparisons between sexes within each interval category based on 95% credible intervals of contrasts between estimated marginal means.

Dependent Variables	Interval	Stallion (S)–Mare (M)	Stallion (S)–Gelding (G)	Mare (M)–Gelding (G)
Rank	≤1 year	* S < M	* S < G	M = G
Rank	2 years	S = M	S = G	M = G
Rank	3 years	S = M	S = G	M = G
Rank	NI	S = M	S = G	* M < G
Round time 1	≤1 year	S = M	S = G	M = G
Round time 1	2 years	S = M	S = G	* M > G
Round time 1	3 years	S = M	S = G	M = G
Round time 1	NI	S = M	S = G	M = G
Round time 2	≤1 year	* S < M	* S < G	* M > G
Round time 2	2 years	S = M	* S > G	* M > G
Round time 2	3 years	S = M	S = G	M = G
Round time 2	NI	S = M	S = G	M = G
Obstacle faults 1	≤1 year	* S < M	* S < G	M = G
Obstacle faults 1	2 years	S = M	S = G	M = G
Obstacle faults 1	3 years	S = M	S = G	M = G
Obstacle faults 1	NI	S = M	S = G	M = G
Obstacle faults 2	≤1 year	* S < M	* S < G	M = G
Obstacle faults 2	2 years	S = M	S = G	M = G
Obstacle faults 2	3 years	S = M	S = G	M = G
Obstacle faults 2	NI	S = M	S = G	M = G

An asterisk (*) denotes a statistically significant difference. Equality (=) and inequality symbols (<, >) indicate which sex showed superior performance.

**Table 2:** Pairwise comparisons between interval categories within each sex based on 95% credible intervals of contrasts between estimated marginal means.

Dependent Variables	Sex	≤1 Year–2 Years	2 Years–3 Years	≤1 Year–3 Years
Rank	Stallion	*	NS	*
Rank	Mare	*	NS	*
Rank	Gelding	*	*	*
Round time 1	Stallion	NS	NS	NS
Round time 1	Mare	*	NS	NS
Round time 1	Gelding	NS	NS	*
Round time 2	Stallion	*	NS	*
Round time 2	Mare	*	NS	*
Round time 2	Gelding	*	*	*
Obstacle faults 1	Stallion	*	NS	*
Obstacle faults 1	Mare	NS	NS	NS
Obstacle faults 1	Gelding	*	NS	*
Obstacle faults 2	Stallion	*	NS	NS
Obstacle faults 2	Mare	NS	NS	NS
Obstacle faults 2	Gelding	*	*	*

An asterisk (*) denotes a statistically significant difference. NS indicates no statistically significant difference. Comparisons between each interval category and NI were not performed.

**Table 3:** Conditional and marginal R^2^ values for outcome variables.

Dependent Variable	Conditional R^2^	Marginal R^2^
Transformed rank	0.44 (95% CI [0.39–0.48])	0.07 (95% CI [0.05–0.09])
Round time 1	0.45 (95% CI [0.41–0.49])	0.02 (95% CI [0.01–0.03])
Round time 2	0.65 (95% CI [0.62–0.67])	0.04 (95% CI [0.03–0.05])
Obstacle faults 1	0.40 (95% CI [0.31–0.49])	0.07 (95% CI [0.04–0.10])
Obstacle faults 2	0.41 (95% CI [0.30–0.51])	0.06 (95% CI [0.04–0.09])

## Data Availability

The competition records are publicly accessible through the Retired Racehorse Cup (RRC) website (https://jouba.nrca.or.jp/rrc/; accessed on 6 February 2026). The raw data (Excel format) were provided by the National Riding Club Association of Japan with permission for research use and publication. The authors curated and compiled the analysis dataset, which is provided as Supplementary Table S1. The statistical model specifications and R scripts are available from the corresponding author upon reasonable request.

## Supplementary Materials

The following supporting information can be downloaded at https://www.mdpi.com/article/10.3390/ani16040562/s1, The Excel version of the RRC competition records used in this study is provided as Table S1. The levels of the random-effect factors are provided as Table S2. Posterior predictive interval (PPI) coverage for each dependent variable (transformed rank, round time 1, round time 2, obstacle faults 1, and obstacle faults 2) is provided in Table S3. Descriptive statistics (median [min–max]) of performance outcomes (transformed rank, round time 1, round time 2, obstacle faults 1, and obstacle faults 2), stratified by sex, interval, age, and number of competitions, are provided in Table S4. Figure S1. Descriptive analysis of performance in show-jumping competitions for retired Thoroughbred racehorses. (A) Proportions of horses, riders, and rider–horse pairs according to the number of competitions in which they participated. (B) Proportions of horses, riders, and rider–horse pairs that competed across multiple years. “Same year” indicates participation multiple times within a single year, whereas “Different 2–7 years” indicates participation across 2 to 7 different years. (C) Number of horses participating in each competition year. (D) Mean age (mean ± SD) of horses in each competition year. (E) Mean rank (mean ± SD) in each competition year. (F) Mean number of horses participating in each interval category (mean ± SD). In panels (C–F), solid lines and filled circles indicate annual trends and means for each competition year, respectively, and red, green, and blue bars represent stallions, mares, and geldings, respectively.
