# Supplementary figures and images for: Sex-Related Differences in Show-Jumping Performance of Retired Thoroughbred Racehorses in Relation to the Interval Since Race Retirement
Source: Animals (Basel). 2026 Feb 11;16(4):562. doi: 10.3390/ani16040562 (PMC12937418; doi:10.3390/ani16040562)

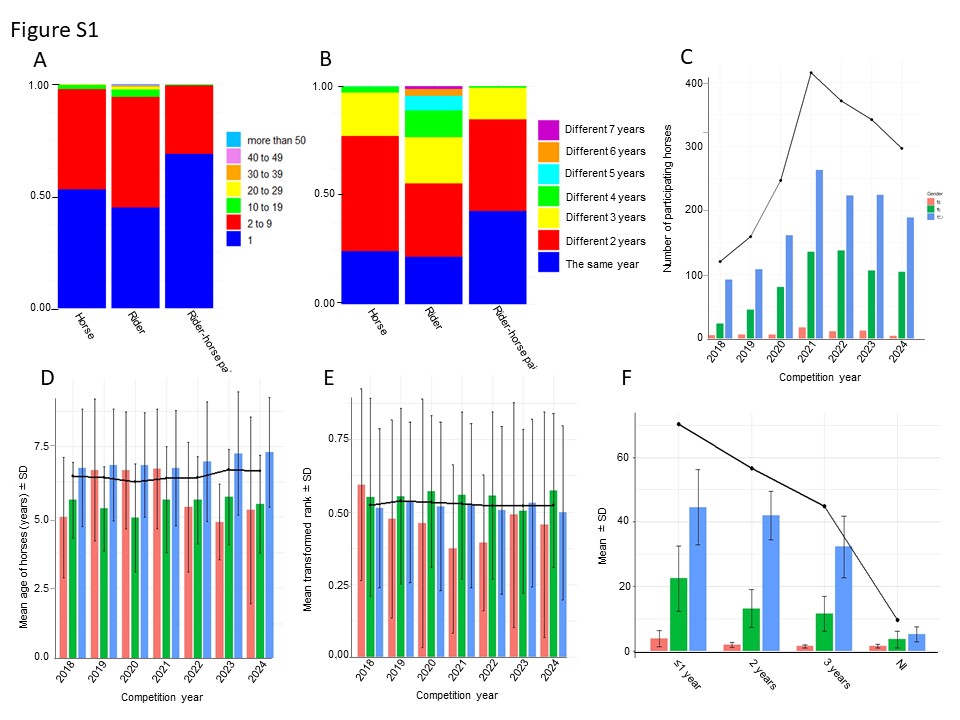

Supplement: Supplementary file 1 [file animals-16-00562-s001.zip › animals-4104398-Figure S1.jpg]
